# Exploring fetal skeletal alterations induced by gold nanoparticles in mice confirmed by laser speckle imaging and LIBS approach

**DOI:** 10.1186/s13062-026-00742-2

**Published:** 2026-03-13

**Authors:** Shaimaa M. I. Alexeree, Doaa Youssef, Ahmed H. Galmed, Ragaa M. Elbalshy, M. Abdel-Harith

**Affiliations:** 1 Department of Laser Applications on Metrology, Photochemistry, and Agriculture, National Institute of Laser Enhanced Science, Cairo University, Giza, 12613 Egypt; 2 Department of Engineering Applications of Lasers, National Institute of Laser Enhanced Science, Cairo University, Giza, 12613 Egypt; 3 Department of Zoology, Faculty of Science, Benha University, Benha, 13511 Egypt; 4 National Institute of Laser Enhanced Science, Cairo University, Giza, 12613 Egypt

**Keywords:** Metal nanoparticles, Transplacental transfer, Nanotoxicology, LIBS, Laser speckle imaging, Osteological

## Abstract

**Background:**

The rapid advancement of nanotechnology has expanded the use of nanomaterials across biomedical and industrial applications. Engineered gold nanoparticles (Au NPs) have attracted considerable interest for diagnostic and therapeutic applications; however, their ability to cross the placental barrier and the potential for fetotoxic effects remain insufficiently explored.

**Results:**

This study aimed to investigate the transplacental transfer and developmental toxicity of biosynthesized Au NPs in a BALB/c mouse model. Pregnant mice were assigned to three groups: a control group (G1) and two treatment groups receiving intravenous doses of 10 µg/g/day (G2) or 20 µg/g/day (G3). Morphological examination of fetal skeletal structures using light microscopy revealed no overt abnormalities. In contrast, comprehensive skeletal assessment using an advanced multimodal complementary laser-based platform demonstrated significant dose-dependent alterations. Laser-Induced Breakdown Spectroscopy (LIBS) detected pronounced dysregulation of calcium and magnesium critical for bone mineralization. Additionally, laser speckle imaging enabled sensitive, nondestructive evaluation of microstructural changes associated with fetal bone ossification and alterations in mineral content. The integrated analysis revealed disrupted ossification centers, abnormal bone density signatures, and subtle skeletal anomalies that were undetectable by conventional microscopy.

**Conclusions:**

The combined application of LIBS and laser speckle imaging proved highly sensitive in identifying early elemental imbalances and microstructural defects in fetal bone development following Au NPs exposure. These findings emphasize the value of advanced photonic and spectroscopic techniques for nanosafety assessment and underscore the necessity for thorough in vivo evaluation of the potential developmental risks associated with biosynthesized gold nanoparticles.

## Background

Nanomaterials have emerged as essential agents in modern bioscience due to their unique optical and surface properties at the nanoscale. Enhanced photoreactivity, quantum confinement, large surface-area-to-volume ratios, and high tunability are among the behaviors of nanomaterials that are not seen in their bulk state. Thus, these qualities make nanomaterials ideal for interacting with biological systems in a precise, highly controlled manner. Overall, Nanoparticles (NPs) can be synthesized through physical, chemical, and biological methods. However, physical and chemical approaches offer good control over particle properties, they may require high energy inputs or use of harmful materials. Conversely, green synthesis uses plant extracts or microorganisms as natural reducing and stabilizing agents, enabling NPs formation under mild, environmentally friendly conditions [1, 2]. It is also important to distinguish these essential synthesis pathways from industrial activities, which may release incidental NPs or ultrafine particles as unexpected byproducts. Unlike such incidental emissions, engineered NPs are produced only through purpose-designed, controlled synthesis processes. Regardless of NPs’ sources and uses, they can enter the body through ingestion, inhalation, skin uptake, or direct injection into blood vessels and accumulate in tissues as foreign bodies [3–5].

Besides, NPs often exhibit greater biological reactivity and potential toxicity than their larger counterparts. Their small size promotes deep pulmonary deposition and prolonged alveolar retention, facilitating translocation across the air-blood barrier into systemic circulation. The markedly higher surface-to-mass ratio of NPs enhances surface reactivity. It can accelerate dissolution or ion release compared with bulk particles, thereby increasing the likelihood of biochemical interactions, such as oxidative stress and inflammatory responses. These unique physicochemical behaviors underscore the need for rigorous in *vivo* investigations to characterize the biodistribution, persistence, and dose-dependent biological effects of nanoparticles [6]. Upon exposure to biological fluids, NPs rapidly adsorb biomolecules onto their surfaces, forming a protein corona that determines their biological identity. The composition of this protein is dependent on the individual components’ concentration and their affinities toward the surface of NPs. Following cellular interaction, NPs are encapsulated in vesicles and transported into and out of the cells during endocytosis and exocytosis. Moreover, NPs may enter cells by passive diffusion across the cell membrane [7].

Despite the widespread use of NPs in many biological and medical applications, there is no clear data on their tissue bioavailability and in vivo toxicity. Although Au NPs have been recognized as nontoxic in some reports [8], several in vitro studies show that they can exhibit cytotoxic effects at relatively high concentrations. This toxicity depends on the shape and physical properties of Gold Nanoparticles (Au NPs) [9]. Nevertheless, there are a few available in vivo studies in the literature [10]. Females are more sensitive to the various effects of NPs. The toxicity of females may lead to decay of fertility and fetal development. NPs are found to cause adverse effects on the reproductive organs as they can penetrate via biological barriers. Fetuses are known to be more sensitive to environmental toxins than adults. It is pointed out that many chemical toxins in the water, air, and food may induce pregnancy complications. Several studies have reported transplacental transfer of NPs during pregnancy, as well as induction of neurotoxicity in their offspring [11]. In addition, exposure to titanium oxide nanoparticles (TiO2 NPs) with a diameter of about 35 nm has been shown to affect pregnant animals, leading to embryo resorption and reduced fetal growth [12].

During embryonic development, Early bone mineralization is fundamentally based on NPs, which are also essential for determining bone hardness. The initial stages of skeletal formation depend on the controlled deposition of nanoscale hydroxyapatite crystals within a collagen matrix. This leads to the production of the first mineralized zones, which subsequently mature into stronger bone tissue. Engineered NPs, such as metal NPs, nano-hydroxyapatite, calcium phosphate nanocrystals, and metal oxide NPs, can modify mineral deposition and collagen position. Moreover, this potentially influences the strength and hardness of embryonic bone. Thus, the nanoscale framework highlights the crucial connection between NPs organization and the structural integrity of embryonic bone [13, 14]. Therefore, it is crucial to study the effects of NPs on pregnant mice.

Nondestructive optical techniques play a crucial role in biological and biomedical research. They can enable rapid, minimal invasive, and noncontact characterization of tissue without compromising sample integrity [15]. Among these techniques, atomic spectroscopy, known as laser-induced breakdown spectroscopy (LIBS), offers ease of use and multi-elemental analysis with little or no sample preparation [16]. It allows sensitive detection of elemental composition in complex biological samples. One of the most recent applications of LIBS is hardness estimation. This approach was first introduced by Tsuyuki et al. [17], who observed a relation between the surface hardness of concrete and calcium ionic-to-atomic LIBS spectral line ratios. Subsequently, Khalil et al. [18] applied LIBS to estimate the hardness of zeolite samples. Following these investigations, Yahiaoui et al. [19] verified the relationship between the surface hardness of α-alumina ceramics and different LIBS spectroscopic parameters, including the ionic-to-atomic line intensity ratio and excitation temperature. These findings were further supported by Galmed et al. [20–22], who confirmed a strong relationship between LIBS diagnostic parameters and surface hardness.

Complementarily, laser speckle imaging has emerged as a powerful label-free, nondestructive optical modality for probing microstructure and dynamical properties of biological tissues. The granular intensity pattern, called speckle, that arises from the interference of scattered waves when a diffuse medium is illuminated by coherent light encodes significant information about the underlying structure. Speckle-based methods have been applied across a broad range of biomedical applications. It has been extensively used for blood flow monitoring in neuroscience and microcirculation [23, 24]. Speckle contrast has been applied to map microvascular remodeling over time in vivo and to characterize wound healing [25, 26]. It is desirable for developmental and toxicological studies [15, 27, 28]. Moreover, speckle analyses have been employed to probe structural alterations in cartilage degeneration [29, 30]. These diverse applications highlight the versatility of speckle imaging. Meanwhile, at the microscale, speckle statistics offer a distinct fingerprint of tissue morphology, as they are susceptible to variations in the scattering centers [31].

The main objective of this study is to investigate the functional necessity of green-synthesized Au NPs on the morphology of treated adult pregnant mice and their fetuses. In addition, we examine the effects of Au NPs on fetal osteology. The osteology effect is confirmed by using two complementary techniques. First, LIBS is employed to assess bone mineral content through elemental analysis. Second, we introduce a speckle microstructure index (SMI), a quantitative parameter derived from speckle pattern statistics, to characterize optical microstructural variations in the fetuses’ skeletal system. SMI provides a nondestructive optical metric that correlates with bone ossification and mineral content changes induced by Au NPs treatment.

## Materials and methods

### Materials

All solutions of reaction materials were prepared in deionized distilled (d.d.) water obtained from a Milli-Q water purification system. All glassware was washed with aqua regia (HCl: HNO_3_ = 3:1 (v/v)) and then rinsed with deionized water. Fresh *(Solanum tuberosum)* was purchased from the local market. Chloroauric acid (HAuCl_4_), and sodium hydroxide (NaOH) were purchased from Sigma-Aldrich (St. Louis, Missouri, USA). The BALB/c mice *(Mus musculus)* were purchased from *the* National Cancer Institute, Cairo University, Egypt.

### Synthesis of Au NPs

The biosynthesis of Au NPs was performed using a simple method with potato extract, as described elsewhere [1].

### Experimental animals

The present investigation was conducted using a pure strain of BALB/c mice *(Mus musculus)*. This strain is characterized by high fertility, a short gestation of about 21 days, a high litter size, genetic stability, and availability in most laboratories. The adult virgin females of approximately age 2–3 months (20–25 g body weight provided) were selected, and each of them was mated overnight with a single fertile male (24–30 g body weight) of the same strain. The first day of pregnancy was assured by the presence of a vaginal plug, or by taking a drop from the vaginal contents and examining for the presence of spermatozoa. These females were separated and used for the experiments. The used mice had free access to food and water and were maintained on a 12 h dark/light cycle in a room with controlled temperature (25 ± 2 °C). All experiments using live animals were carried out with approval from the Cairo University Institutional Care and Use Committee **(CU-IACUC)** based on reviewing the application number **CU/I/F/41/18.**

#### Grouping of mice

Eighteen pregnant female mice were randomly divided into three groups: one control group and two experimental groups treated with different doses of Au NPs. Each group has six mice. Mice received intravenous (IV) injections (via a tail vein) of approximately 100 µL of Au NPs solution (adjusting the final volume with the animal weight for the given dose) at doses of 10 and 20 µg/g daily for 7 days, starting from the 5th day of gestation [32].

##### Group one (G1)

The control group (negative control) was of the same inbred strain as the treated animals.

##### Group two (G2)

The experimental mice group was treated intravenously with 100 µL of Au NPs at 10 µg/g/day for 7 days.

##### Group three (G3)

The experimental mice group was treated intravenously with 100 µL of Au NPs at 20 µg/g/day for 7 days.

The pregnant females were dissected on the 20^th^ day of gestation. The uteri were removed by cesarean section, and fetuses were collected.

#### Teratological examination

All living fetuses were divided into two groups for Morphological and Osteological studies. The studies were carried out on maternally treated fetuses on the 20^th^ day of gestation to determine the teratogenic effects of Au NPs.

#### Morphological studies

The weight of control and Au NPs-treated pregnant mice, and the percentages of fetal survival in all groups, were recorded at the end of the experiment (20^th^ day of gestation). The percentage of body weight gain was calculated. The weight of the carcass (the pregnant uterus and the animal’s body weight without the pregnant uterus) was also recorded. The pregnant females of all groups were sacrificed on the morning of the 20^th^ day of gestation. The lengths and weights of the 20-day fetuses were recorded. The total number of fetuses, either living or dead, was counted. Additionally, morphological examinations for any external malformation were studied.

#### Skeletal malformations

For osteological examinations, fetuses were prepared for skeletal analysis by using Alizarin red-S staining and Alcian blue-E staining techniques. Fresh fetuses were fixed in 95% ethyl alcohol for 5 days, and then they were put in acetone for 2 days. The fetuses were stained for 3 days in 20 mL staining solution at 37 °C. The staining solution is composed of 1mL of 0.1% Alizarin red-S in 95% ethyl alcohol, 1 mL of 0.3% Alcian blue-E in 70% ethyl alcohol, 1 mL of glacial acetic acid, and 17 mL of 70% ethyl alcohol. The staining specimens were examined under the dissecting binocular microscope to study the various parts of the axial and appendicular skeleton for any malformation [33].

### Experimental setup of LIBS

An Nd: YAG laser (BRIO, Quantel, France) of 1064 nm wavelength, 35 mJ pulse energy, and 6 ns laser pulse duration was used to generate the LIBS experiment. A lens with a 10 cm focal length was used to focus the laser beam onto the target surface, which was mounted on a horizontally movable stage. The distance between the sample and the lens was adjusted to 1 mm before the focal point to ensure no air breakdown occurs. A 0.6 mm aperture fiber optic was used to collect plasma emission, which was fed into an Echelle spectrometer coupled with an ICCD camera (Mechelle 7500 with PCO ICCD camera). The angle (45°) and the distance between the optical fiber and the sample were adjusted to ensure that the plasma is within the fiber aperture’s collection cone and that the signal-to-noise ratio is optimal. The camera’s optimal triggering conditions were tested with a 1.5 µs delay and a 10 µs gate width. Each sample spectrum was recorded by averaging five spectra at different positions on the sample surface; each of these spectra is the accumulation of 10 single-shot spectra. This is done to reduce the laser shot-to-shot fluctuations and to increase the signal-to-noise ratio. The data were then analyzed using LIBS + + software [34].

### Laser speckle imaging and analysis

#### Imaging setup

*Ex* vivo laser speckle imaging was performed to estimate the optical microstructure characteristics of fetuses’ skeletal systems collected at the end of the 20^th^ day of gestation. The skeletal systems were placed on a vibration-isolated optical table and on a matte black background to reduce back reflections. A continuous-wave 633 nm, 5 mW He-Ne laser was expanded through collimating lenses to form a 6 mm uniform illumination spot on the bone surface. The diffusely reflected speckle patterns were captured by a CMOS camera (4608 × 3456 pixels) mounted 60 cm from the bone at a small off-axis angle to reduce specular reflection. Further information is available elsewhere [35].

#### Statistical and spectral analysis

The speckle distributions were analyzed to extract three quantitative metrics that capture distinct characteristics associated with ossification and mineral content of the underlying bone microstructure: global contrast ($\:K$), entropy ($\:H$), and Power Spectral Density (PSD) slope ($\:\beta\:$). The global speckle contrast is computed as the ratio between the standard deviation ($\:\sigma\:$) and the mean ($\:\mu\:$) of the intensity values of the entire image as follows [26]:

1 $$\:K=\:\frac{\sigma\:}{\mu\:}$$

This metric was applied to represent the overall speckle variability across the image and serves as an indicator of scattering heterogeneity. A low contrast value reflects increased optical uniformity and reduced scattering heterogeneity, which are associated with higher mineral density and more advanced ossification.

The entropy was computed using the Shannon entropy of the normalized intensity histogram (Eq. (2)). Shannon entropy is frequently used to analyze complex biological and physical systems because it captures linear dynamics and stochastic variability [36]. In this study, it was applied to capture the statistical complexity of the speckles’ intensity distribution arising from the heterogeneous microstructure and irregular scattering interface. Reduced entropy values suggest organized, compact microstructures, driven by stronger, more coherent backscattering radiation from mineralized regions. In this context, the entropy metric can serve as a sensitive measure of microstructural organization and ossification progression in developing bone tissue.

2 $$H = - \sum\limits_{i = 1}^L {{p_i}\>lo{g_2}\left( {{p_i}} \right)}$$

where $\:{p}_{i}$ is the normalization probability of the $\:{i}^{th}$ gray-level intensity and $\:L$ is the total number of intensity levels in the speckle pattern.

The PSD provides a frequency-domain representation of the spatial intensity variations in the image, reflecting the underlying texture and structural organization. Herein, PSD is computed by applying a 2D Fourier transform of the speckle image $\:I(x,y)$ as follows:

3 $$\:PSD\left({f}_{x},{f}_{y}\right)=\:{\left|F\left\{I(x,y)\right\}\right|}^{2}$$

where $\:{f}_{x}$ and $\:{f}_{y}$ These are the spatial frequency components. Then, PSD is radially averaged to obtain a one-dimensional spectrum $\:P\left(f\right)$, which represents the average power as a function of spatial frequency. $\:P\left(f\right)$ exhibits a power law behavior as follows [37]:

4 $$\:P\left(f\right)\propto\:{f}^{-\beta\:}$$

The PSD slope ($\:\beta\:$) was then employed in this study to characterize the speckle grain organization related to the degree of ossification and mineralization. For instance, as bone matrix becomes denser and more homogeneous during development, the speckle pattern shows reduced high-frequency components and larger structural domains, leading to a steeper slope (an increase in the $\:\beta\:$ value).

#### Speckle microstructure index

To integrate the optical information from the three metrics ($\:K$, $\:H$, and $\:\beta\:$), which are related to bone microstructure, we propose a Speckle Microstructure Index (SMI) as a composite quantitative nondestructive optical parameter. Since $\:K$and $\:H$ are inversely related to structure uniformity and mineralization, the SMI was computed as a weighted linear combination as follows:

5 $$\:SMI={w}_{K}\left(1-K\right)+{w}_{H}\left(1-H\right)+{w}_{\beta\:}\beta\:$$

where each metric was normalized to [0,1], and $\:{w}_{K}$, $\:{w}_{H}$, and $\:{w}_{\beta\:}$ represent the corresponding weighting coefficients. These coefficients reflect the relative contribution of each metric. Therefore, the SMI value was defined as increasing with ossification and mineralization, ranging from optically soft (low SMI) to hard (high SMI) bone regions.

### Statistical analysis and visualization

The effect of Au NPs on the (+) or (-) obtained values is calculated as a percentage change from the control. Statistical analysis was performed using one-way analysis of variance (ANOVA), and significance was determined at *p* < 0.05.

Regarding laser speckles, the SMI values were compared across the different experimental groups using mean ± standard deviation and Kernel Density Estimation (KDE). The KDE provides a nonparametric estimate of the SMI probability density function, allowing a smooth representation of its distribution. The relationship between the three metrics, as well as SMI values, and the ossification level was measured using the Spearman correlation coefficient ($\:\rho\:$).

## Results

### Au NPs characterization

**Fig. 1:**
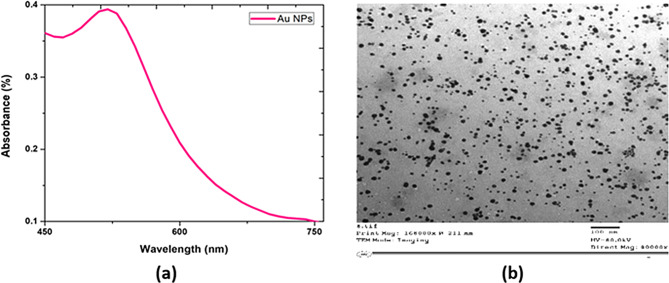
(**a**) UV–Vis spectra of Biosynthesized Au NPs prepared from potato extract with the characteristic peak at about 520 nm. (**b**) a TEM image of biosynthesized Au NPs with an average size of approximately 12 nm (scale bar = 100 nm)

Figure 1(a) illustrates the UV–Vis absorption spectrum of the biosynthesized Au NPs in an aqueous solution. Au NPs have a characteristic absorption peak at 520 nm assigned to the Surface Plasmon Resonance (SPR) band. Additionally, Fig. 1(b) indicates the Transmission Electron Microscopy (TEM) of biosynthesized Au NPs from potato extract. This image explores their spherical shape with an average size of about 12 nm.

### Osteological observations

**Fig. 2:**
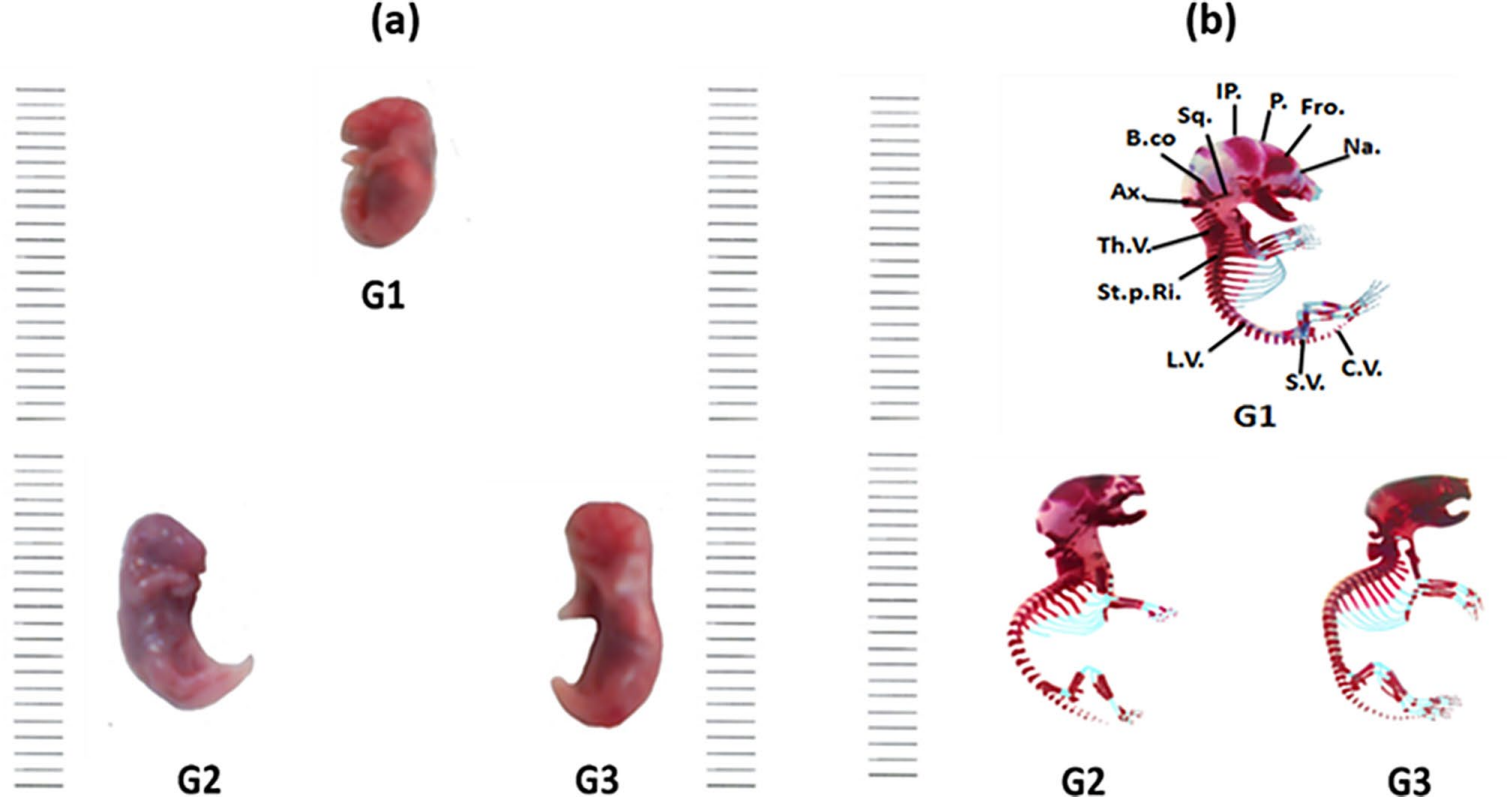
(**a**) Photographs of fetuses on the 20^th^ day of gestation for a control [G1] group and the two Au NPs treated groups [G2 (10 µg/g/day for 7 days) and G3 (20 µg/g/day for 7 days)]. (**b**) Photographs of a lateral view of the fetal skeleton of the BALB/c mice on the 20^th^ day of gestation of the control “G1” and the Au NPs treated groups [G2 (10 µg/g/day for 7 days) and G3 (20 µg/g/day for 7 days)]. Abbreviations: Na.: Nasal; Fro.: Frontal; P.: Parietal; IP.: Interparietal; Sq.: Squamosal; B.co.: Basioccipital; Ax.: Axis; Th.V.: Thoracic vertebrae; St.p.Ri: Sternal Portion of Ribs; L.V.: Lumbar Vertebrae; S.V.: Sacral Vertebrae; C.V.: Cervical Vertebrae

The BALB/c mouse skeleton is conventionally divided into two main structures: the **axial skeleton**, comprising the skull, vertebral column, ribs, and sternum, and the **appendicular skeleton**, which includes the pectoral girdle, forelimbs, pelvic girdle, and hind limbs **(**Fig. 2**(b))**. Osteological examination of fetuses from dams treated with varying doses of gold nanoparticles on gestational day 20 revealed pronounced effects on skeletal development. Maternal exposure induced alterations in both chondrogenesis and osteogenesis during the critical stages of skeletal formation, manifesting as changes in ossification patterns and bone morphology.

#### Axial skeleton

##### The skull

**Fig. 3:**
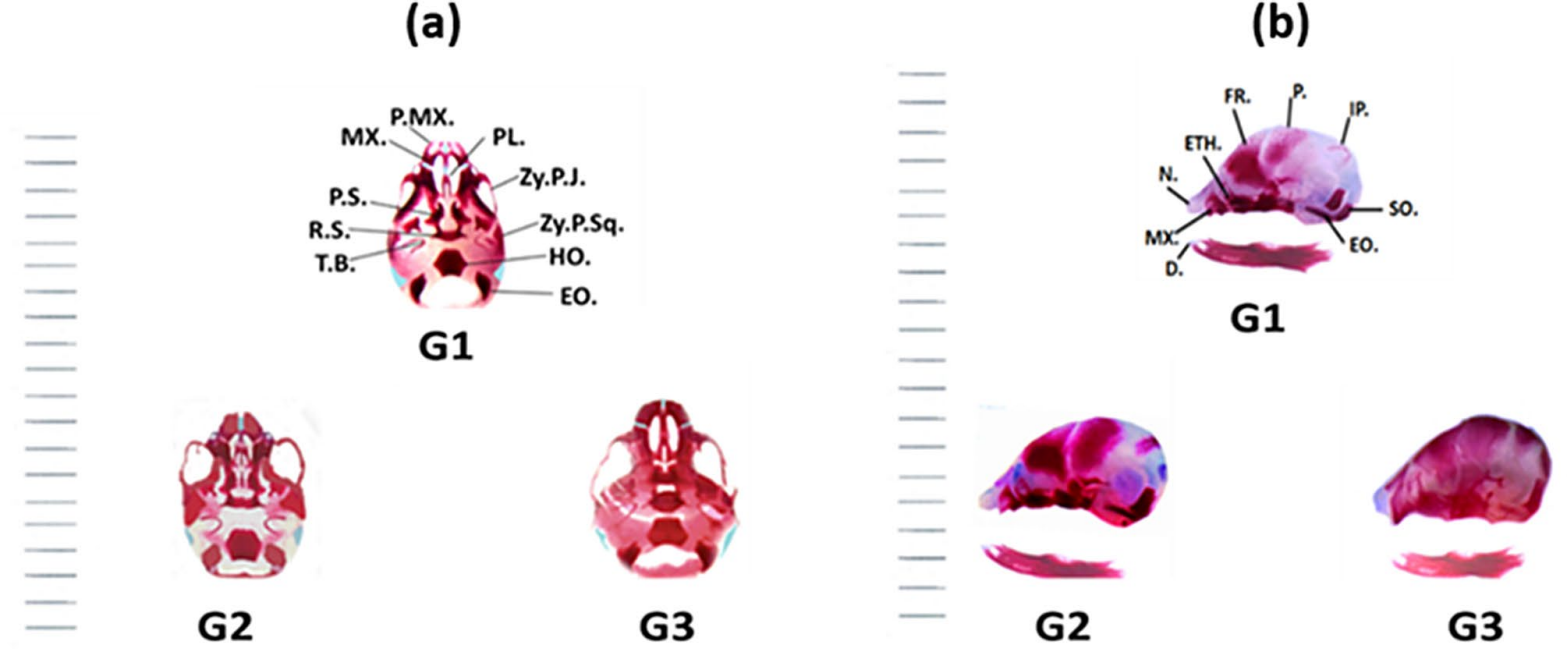
(**a**) Photographs of the ventral view of the skull of the BALB/c mice fetuses of the control “G1” group and the two Au NPs treated groups [G2 (10 µg/g/day for 7 days) and G3 (20 µg/g/day for 7 days)] on the 20^th^ day of gestation. (**b**) Photographs of a lateral view of the skull of the BALB/c mice fetuses of the control “G1” and the two Au NPs treated groups [G2 (10 µg/g/day for 7 days) and G3 (20 µg/g/day for 7 days)] on the 20^th^ day of gestation. Abbreviations: MX.: Maxilla; P.MX.: Premaxilla; PL.: Palatine; P.S.: Presphenoid; BS.: Basisphenoid; T.B.: Tympanic Bulla; Zy.P.J: Zygomatic Process of Jugal; Zy.P.Sq.: Zygomatic Process of Squamosal; EO.: Exoccipital

In the present investigation, the skull of the control fetuses on the 20^th^ day of gestation was in well-ossified conditions. However, the tympanic and squamosal regions are partially ossified. The nasal bone shapes are quadrangular. The intranasal and the front nasal sutures are marked. The frontal bones are paired bones that surround the anterior fontanelle. At the base of the cranium, the ethmoid and presphenoid bones are seen in one plane. The mandible is well ossified with slightly angulated ramus (Fig. 3(a)).

Examination of the fetuses’ skulls maternally treated with “10, and 20 µg/kg” of Au NPs from gestation day 5 for one week, showed moderate to heavy ossification of the skull components compared to the untreated fetuses. Relative to the control, every element of the stained skull indicated that these bones had advanced in development. The skull bones that revealed the same ossification were parietal, interparietal, tympanic bulla, zygomatic process of squamosal, periotic, squamosal, palatine, pterygoid, supraoccipital, and ethmoid bones. The most evident decrement was the dilatation of the bone material, which was minimal in the control group, increased in the second group, and became most advanced in the third group. Moreover, most of the skull components in the third group were stained red with alizarin red-S. This indicated that ossification increased with the higher dose of Au NPs in G3. In addition, the palatal bones showed different degrees of ossification according to the density of the red color of the alizarin dye (Fig. 3(b)).

##### The vertebral column

**Fig. 4:**
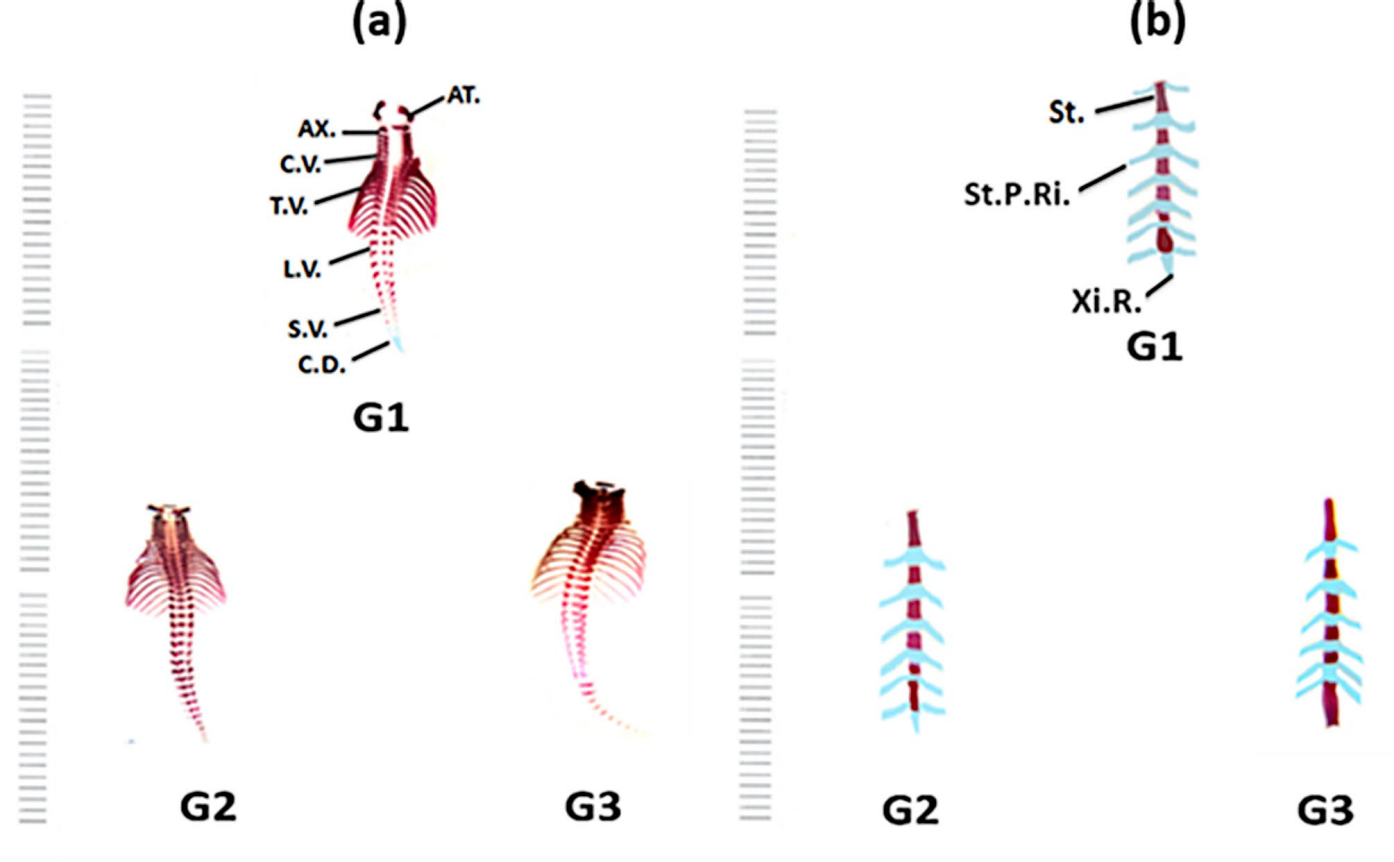
(**a**) Photographs of a ventral view of the vertebral column and the ribs of the BALB/c mice fetuses of the control “G1” group and the Au NPs treated groups [G2 (10 µg/g/day for 7 days) and G3 (20 µg/g/day for 7 days)] on the 20^th^ day of gestation. (**b**) Photographs of a ventral view of the sternebrae ribs of the BALB/c mice fetuses of the control “G1” group and the two Au NPs treated groups [G2 (10 µg/g/day for 7 days) and G3 (20 µg/g/day for 7 days)] on the 20^th^ day of gestation. Abbreviations: AT.: Atlas; AX.: Axis; C.V.: Cervical Vertebrae; T.V.: Thoracic vertebrae; L.V.: Lumbar Vertebrae; S.V.: Sacral Vertebrae; C.D.: Caudal Vertebrate; St.: Sternebrae; St.P.Ri.: Sternal Portion of Ribs; Xi.C.: Xiphoid Cartilage

There are thirty-four segments in the mouse vertebral column, represented as 8 cervical cord segments corresponding to only 7 cervical vertebrae, 13 thoracic, 6 lumbar, 4 sacral, and finally 3 coccygeal with ossified bodies and arches **(**Fig. 4**)**. The repeated, serially symmetric units of the vertebral column perform many functions, such as protecting the spinal cord, bearing weight, providing attachments for musculature, transmitting locomotor power, absorbing shock, and providing support [38].

Examination of the vertebral column of fetuses on the 20^th^ day of gestation, maternally treated with different doses of Au NPs, showed that the atlas and axis vertebrae ossified well when compared to the control. The caudal vertebrae of G2 showed complete ossification, whereas the remaining vertebrae did not differ from the controls. The majority of the G3 fetuses studied had advanced cervical, thoracic, and lumbar ossification and elongation. In addition, sacral and caudal vertebrae were completely ossified in all treated groups compared to the control group.

##### Ribs

The control fetuses on the 20^th^ day of gestation have 13 pairs of ribs with alterations in ossified vertebral portions. Moreover, the sternal portions of the ribs appeared cartilaginous (Fig. 4(a)). The ribs of G2 treated with a 10 µg/g dose of Au NPs showed results like those of the control group. Moreover, the ribs of fetuses in G3 treated with a 20 µg/g dose of Au NPs were longer than those of the control group. The study of the fetuses’ ribs revealed the presence of rib agenesis and thick ribs in response to a high dose of Au NPs (Fig. 4(a)).

##### Sternum

The control fetuses on the 20^th^ day of gestation possess 6 well-ossified sternebrae. The last one of the sternebrae is the xiphisternum. The sternebrae in fetuses maternally treated with different doses of Au NPs were longer than those in the control group. The most affected sternebrae were observed in G3, which received the highest dose of Au NPs (Fig. 4(b)).

#### The appendicular skeleton

##### The pectoral girdle and forelimb

**Fig. 5:**
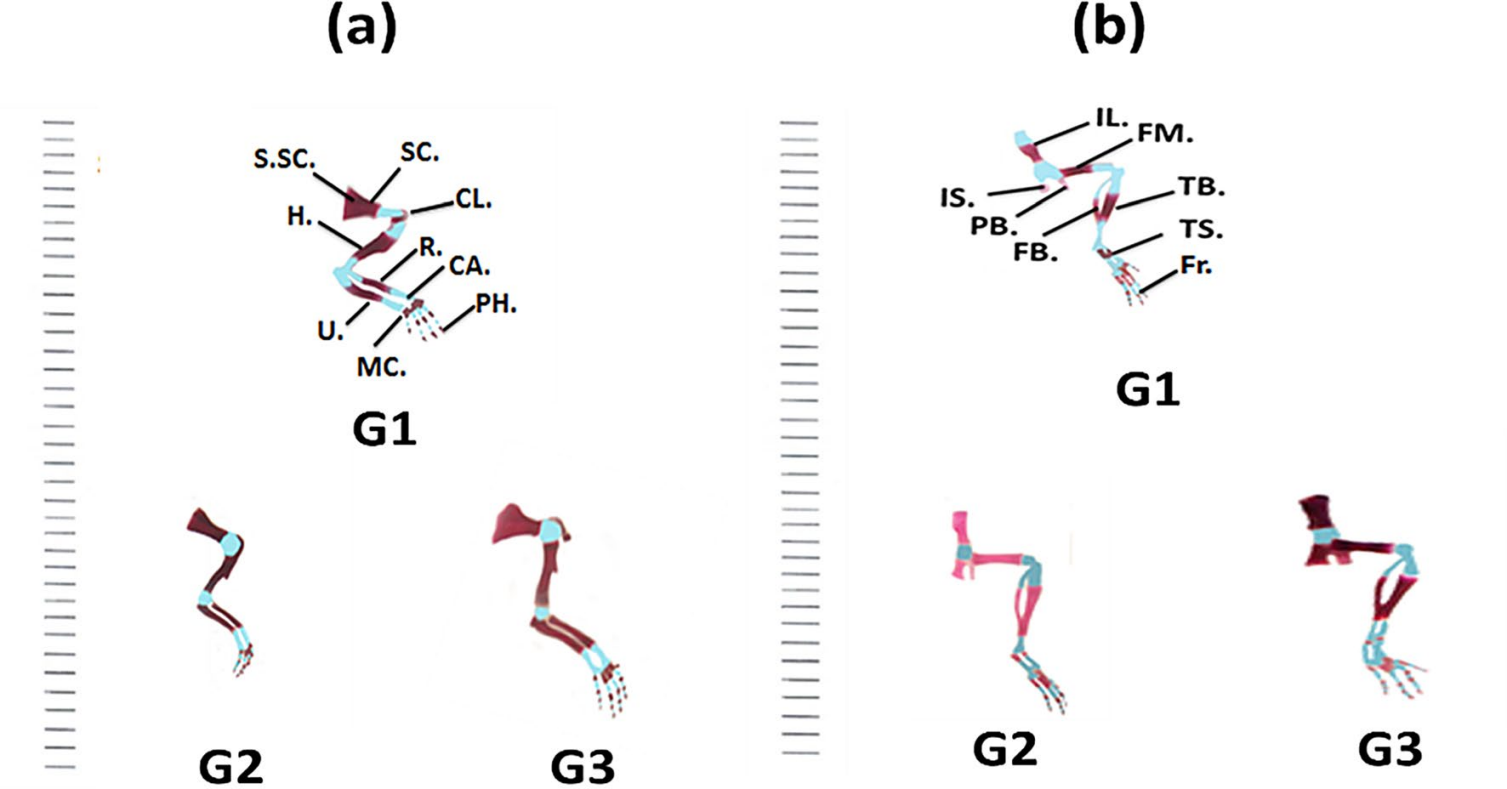
(**a**) Photographs of a lateral view of the pectoral girdle and fore limb of the BALB/c mice fetuses of the control “G1” group and the Au NPs treated groups [G2 (10 µg/g/day for 7 days) and G3 (20 µg/g/day for 7 days)] on the 20^th^ day of gestation. (**b**) Photographs of the lateral view of the pelvic girdle and hind limb of the BALB/c mice fetuses showing: The control “G1” group and the Au NPs treated groups [G2 (10 µg/g/day for 7 days) and G3 (20 µg/g/day for 7 days)] on the 20^th^ day of gestation. Abbreviations: S.SC: Supra-Scapula; SC.: Scapula; CL.: Clavicle; H.: Humerus; R.: Radius. U.: Ulna; CA.: Carpals; MC.: Meta Carpalia; PH.: Phalanges. IL.: Ilium; IS.: Ischium; PB.: Pubis; FM.: Femur; FB.: Fibula; TB.: Tibia; TS.: Tarsia; T: Toes

The control pectoral girdle of the fetuses on the 20^th^ day of gestation is composed of a well-ossified scapula and clavicle. These stained well with Alizarin Red S. However, the suprascapular is still cartilaginous and stained with alcian blue. The forelimb of the fetuses involved an ossified humerus, radius, and ulna, phalanges with five digits, cartilaginous carpalia, and metacarpalia **(**Fig. 5a).

The pectoral girdle and the fore limb of fetuses maternally treated daily with Au NPs from the 5th day of gestation for seven days showed dilation in the degree of ossification compared to the control. At all dose levels of the Au NPs, the pectoral girdle and forelimbs of the examined fetuses of G2 and G3 showed high ossification. The clavicle and scapula bones showed a marked increase in length relative to the control **(**Fig. 5a**)**. This observation is considered a good effect of Au NPs on the fetuses of BALB/c mice during the gestation period.

##### The pelvic girdle and Hind limb

The pelvic girdle of the control fetuses on the 20^th^ day of gestation is composed of three well-ossified bones: ischium (IS), ilium (IL), and pubis (PB). The pubic symphysis remains cartilaginous. The hind limb of the control fetuses involved the well-ossified bones: femur (FM), tibia (T.B.), and fibula (FB), a series of phalanges in the four digits, and cartilaginous tarsals (TS) and metatarsals **(**Fig. 4(b)).

The pelvic girdle and hind limb of fetuses on the 20^th^ day of gestation maternally treated with different doses of Au NPs showed that the degree of ossification of ilium, pubis, ischium, femur, tibia & fibula, and a series of phalanges were affected by such treatment. The ossification was high in the ilium, pubis, ischium, femur, tibia & fibula, and a series of phalanges of the fetuses of the treated groups. The rate of chondrification of the pubic symphysis, tarsals, and metatarsals also increased. The length of the components of the pelvic girdle and fore limb in G3 was longer than in the control group. These results are considered promising for the use of Au NPs during the gestation period **(**Fig. 5(b)).

### Bone ossification through LIBS techniques

**Fig. 6:**
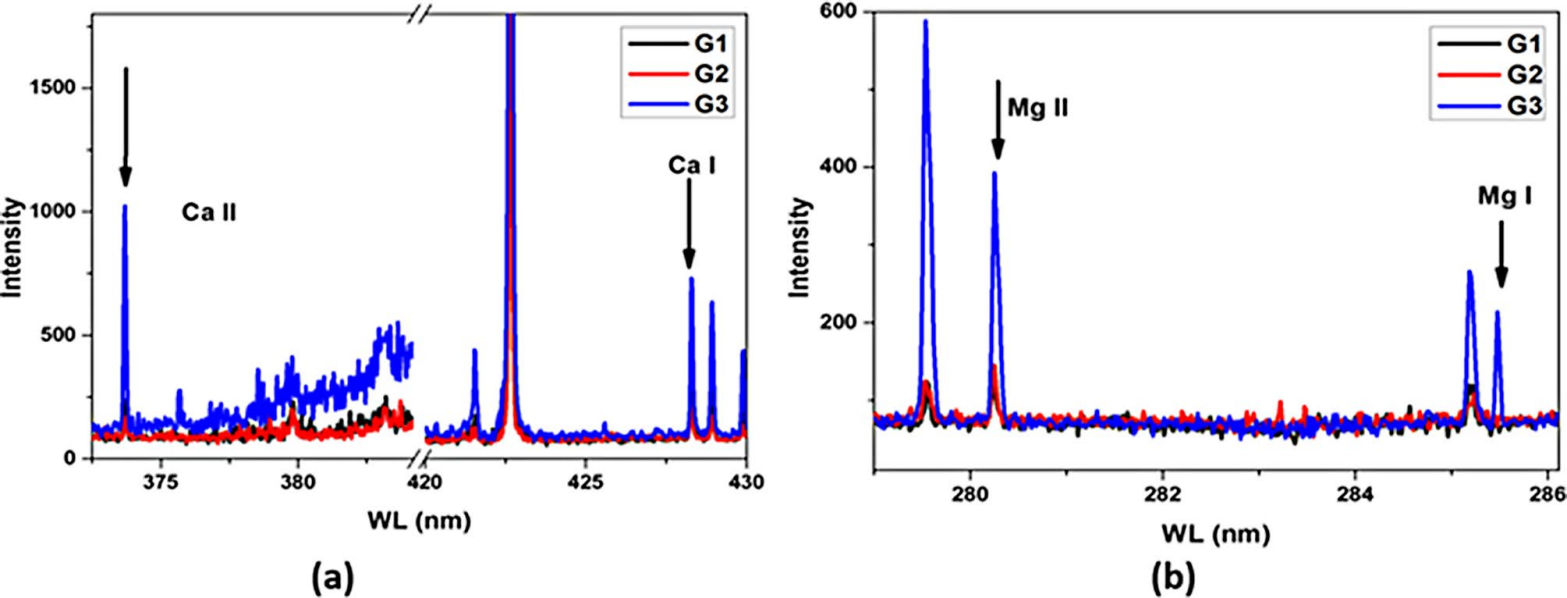
LIBS spectral lines for Ca and Mg used to estimate the level of bone ossification

**Fig. 7:**
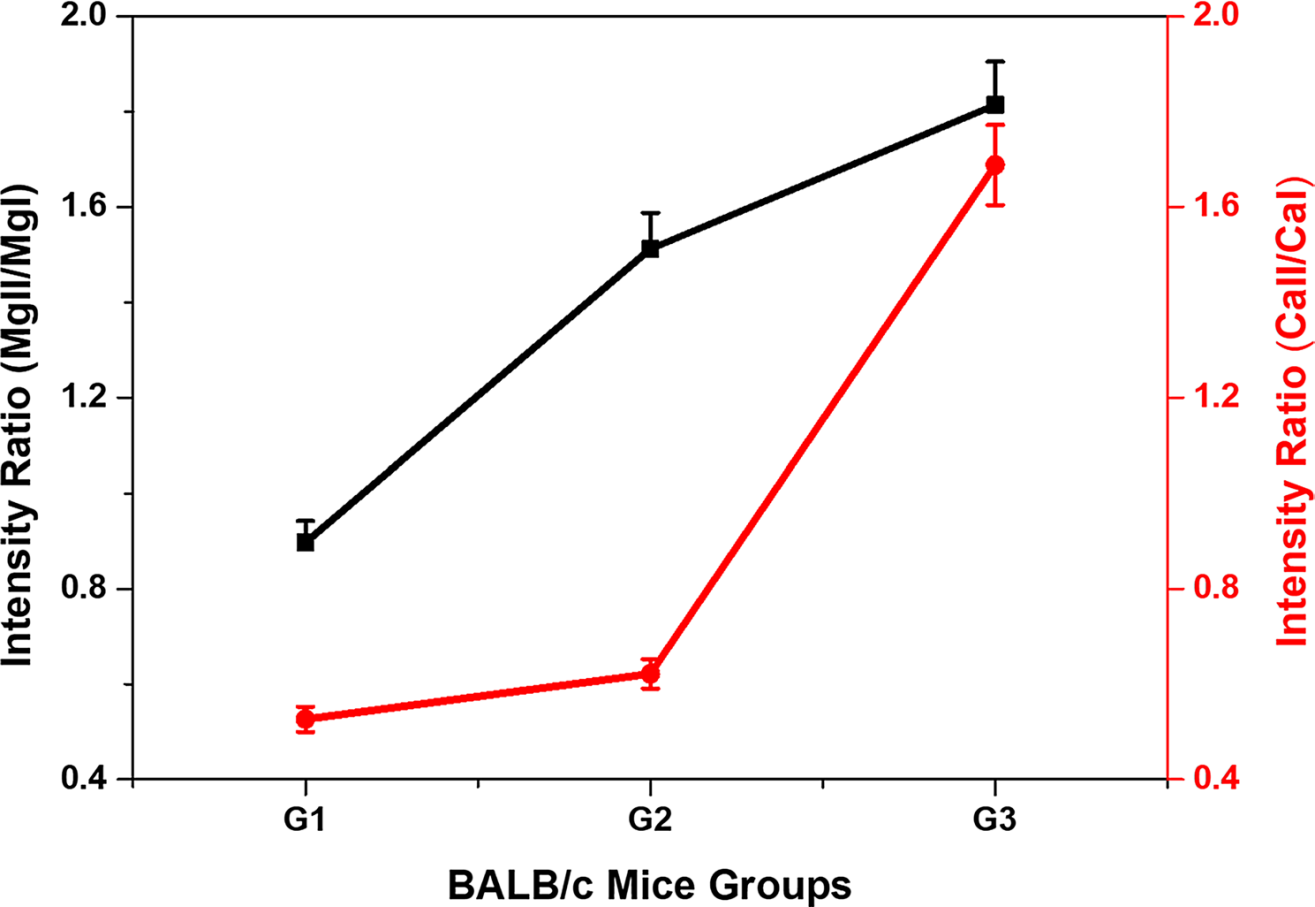
Ionic-to-atomic line intensity ratios of Ca and Mg as measured for bone samples from groups G1, G2, and G3

To estimate the level of ossification of the studied samples, the ionic-to-atomic line ratios of Ca II (373.69 nm) to Ca I (428.9 nm) and Mg II (280.26 nm) to Mg I (285.22 nm) were calculated. Figure 6 shows the LIBS spectral lines used in this analysis for bone samples from groups G1, G2, and G3. The corresponding ionic-to-atomic line-intensity ratios for Ca and Mg are presented in Fig. 7. The results revealed that G1 exhibited the lowest ratios, followed by G2, whereas G3 had the highest ratios. According to the literature [19, 20], these findings indicate an increase in ossification level and mineral density in the high-dose Au NPs treated group.

### Bone ossification through speckle imaging

Bone ossification is a progressive process in which soft cartilaginous tissue is gradually transformed into rigid mineralized bone. As ossification progresses, the bone becomes more homogeneous due to reduced refractive-index fluctuations between the mineral and organic phases. The increase in structural uniformity reduces light-scattering heterogeneity, thereby altering the appearance of laser speckle patterns. Such patterns capture the optical signatures produced by the interference between the scattered light from the bone surface. Since speckle formation is susceptible to surface roughness and submicron variations in refractive index, the resulting texture provides a powerful means of assessing tissue microstructure organization and mineral content.

**Fig. 8:**
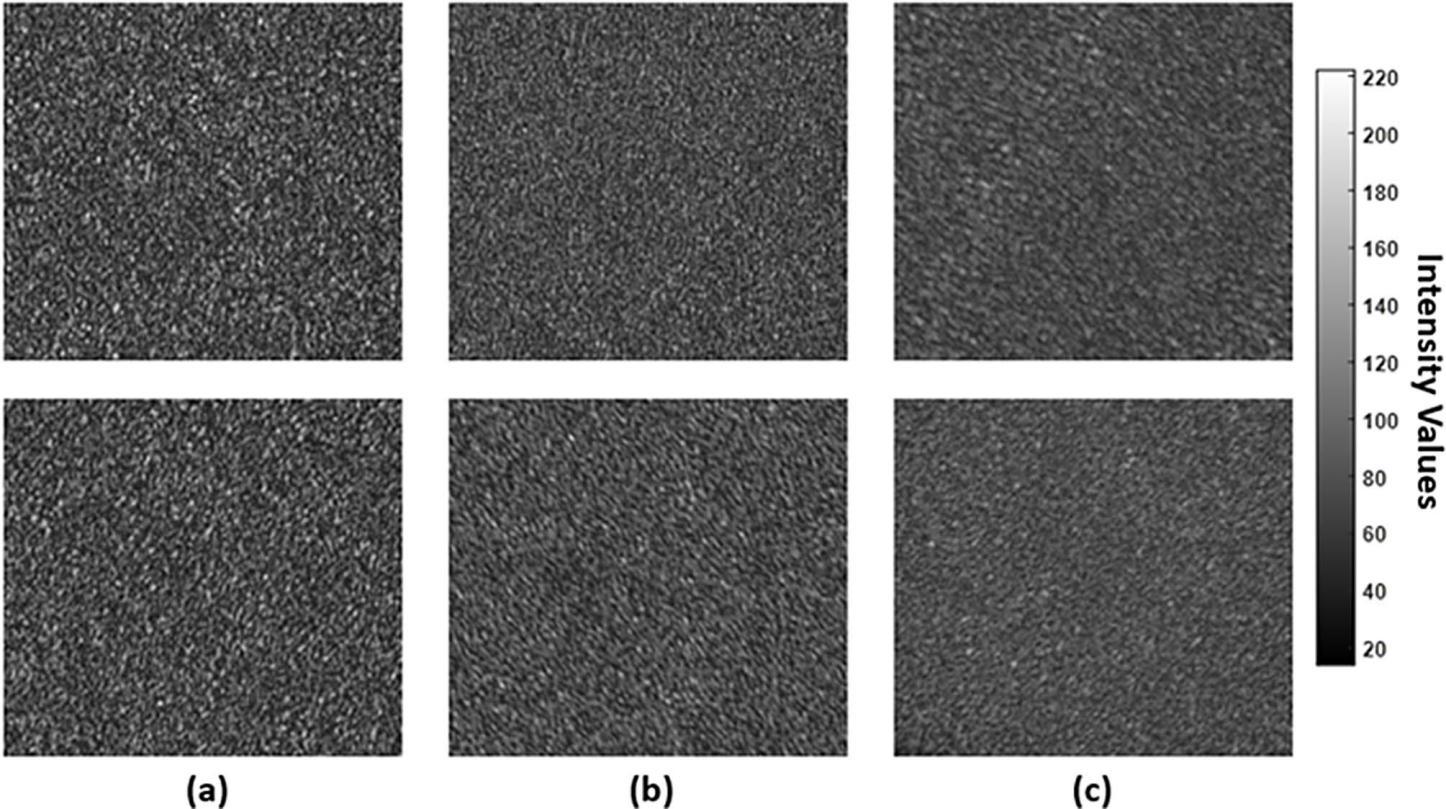
Representative laser speckle patterns recorded from the skull (top) and ribs (bottom) of fetuses’ skeletal system in: (**a**) the control group, (**b**) 10 µg/g Au NPs-treated group, and (**c**) 20 µg/g Au NPs-treated group

To quantitatively estimate the ossification progression of the fetuses’ skeletal system, speckle patterns were acquired from multiple regions of the axial skeleton (including the skull, vertebral column, ribs, and sternum) and the appendicular skeleton (including the pectoral girdle, forelimbs, pelvic girdle, and hind limbs) in all mice. For each group, 80 speckle patterns were collected to evaluate the variability. Figure 8 presents representative raw speckle patterns recorded from the skeletal system of fetuses across experimental groups. These patterns provide structural differences and variations for assessing the ossification development in each group. For instance, the speckle patterns of the control group show coarse, high-contrast speckle grains due to more heterogeneous microstructures and less mineralized tissue. In contrast, the patterns collected from G2 and G3, which were treated with 10 and 20 µg/g doses of Au NPs, respectively, show less contrast and finer speckle textures. These qualitative findings suggest enhanced optical uniformity consistent with advanced ossification and increased mineral deposition. Remarkably, the speckle patterns captured from the fetuses’ skeletal systems encode extremely valuable information about their optical characteristics. These observed variations are consistent with the LIBS results obtained in this study.

**Fig. 9:**
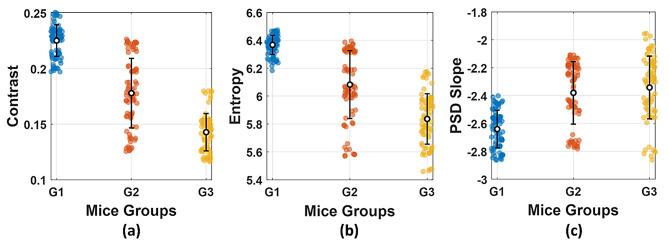
Quantitative analysis of fetal bone speckle patterns across the experimental groups. The metrics include: (**a**) global contrast, (**b**) Shannon entropy, and (**c**) PSD slope. Each point represents one sample. The bars show mean ± standard deviation

For qualitative evaluation of fetal bone microstructural ossification behavior in response to Au NPs treatment, three metrics were computed from the speckle patterns: global contrast, Shannon entropy, and PSD slope. Global contrast reflects the intensity variation in the speckled patterns. Thus, it provides insight into bone structural homogeneity. Shannon entropy quantifies the complexity and randomness of patterns, which, in turn, serves as an indicator of microstructural disorder. The PSD slope characterizes the special frequency content of the speckle patterns, reflecting the organization of speckle grains. Figure 9 shows the computed speckle metrics across the experimental groups. A significant decrease in both global contrast and entropy is observed with increasing the Au NPs dose, generated from greater statistical complexity and homogeneity in the speckle field.

Regarding PSD slope, it becomes steeper in the treated mouse groups, associated with finer optical granularity. These findings represent changes in a well-ossified skeletal system following treatment with Au NPs. It therefore captures information about the scale and distribution of bone microstructures. Thus, it provides a comprehensive assessment of ossification progression and structural alterations.

**Fig. 10:**
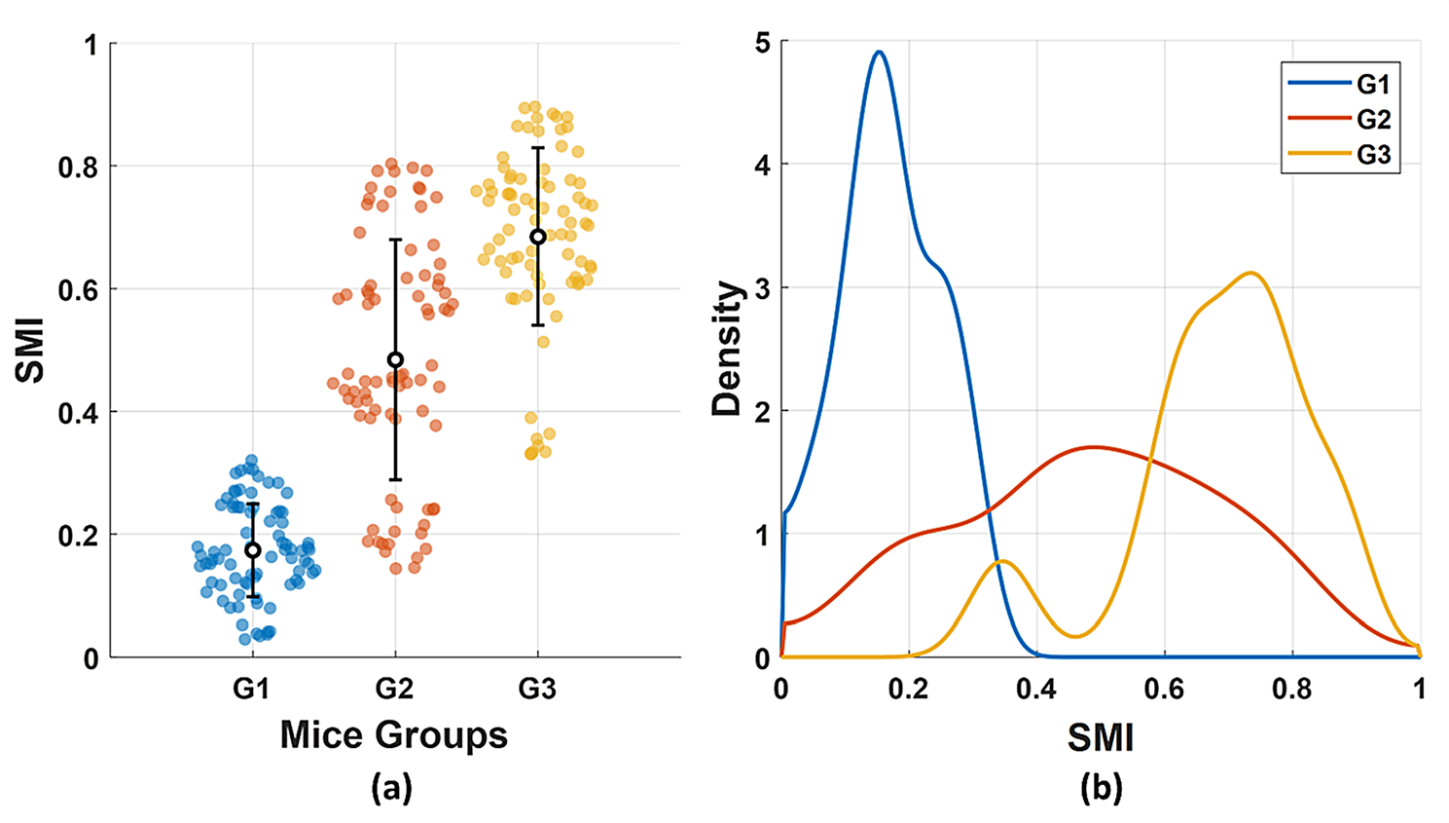
(**a**) Distribution of SMI across experimental groups. (**b**) The kernel density estimation of the SMI values for each experimental group. The bars show mean ± standard deviation

Spearman’s analysis revealed a strong, negative, statistically significant correlation between the global contrast and the ossification level ($\:\rho\:$ = -0.824, *P* < 10^− 10^). Shannon Entropy also presented a strong, negative, statistically significant correlation with a value of $\:\rho\:$ = -0.800, *P* < 10^− 10^. While for the PSD slope, a moderate, positive, statistically significant correlation ($\:\rho\:$ = 0.506, *P* < 10^− 10^) was obtained. Thus, a composite SMI was calculated using Eq. 5 by integrating the global contrast, entropy, and SPD slope into a single measure, along with the corresponding weights. $\:{w}_{K}$, $\:{w}_{H}$, $\:{w}_{\beta\:}$were set to 0.35, 0.35, and 0.30, respectively. SMI showed a strong relationship with the ossification level ($\:\rho\:$ = 0.804, *P* < 10^− 10^), demonstrating its robustness as an optical measure of bone ossification and mineralization. As shown in Fig. 10(a), the mean SMI values increased from the control group to the high-dose Au NPs treated group, confirming an enhancement of the mineral density of the developed skeletal system. The KDE of the SMI values for each experimental group are presented in Fig. 10(b). The control group shows a narrow density peak at lower SMI values. The Au NPs treated groups show right-shifted distributions toward higher values of SMI.

## Discussion

The optical and morphological characterization of the biosynthesized Au NPs provides strong evidence of successful NPs formation using potato extract. This extract was used as a reducing and stabilizing agent. As shown in Fig. 1(a), the UV–Vis absorption spectrum displays a distinct and intense peak at 520 nm, which is characteristic of the SPR of colloidal Au NPs. The presence of this peak confirms the reduction of Au³⁺ ions to metallic Au⁰ nanoparticles. SPR absorption in the range of 515–530 nm is widely reported for spherical gold nanoparticles. This result indicates the formation of well-dispersed NPs with relatively small diameters. Complementing the UV–Vis finding, Fig. 1(b) presents a TEM image of the biosynthesized Au NPs, revealing that Au NPs are predominantly spherical with an average diameter of approximately 12 nm. The small size and uniform morphology observed in the TEM micrograph are consistent with the SPR peak position [1].

Additionally, this supports the correlation between particle size and optical properties. Furthermore, the monodispersed, well-defined spherical shapes suggest that the biomolecules in the potato extract effectively control nucleation and growth during synthesis. Collectively, the UV–Vis and TEM findings validated the successful green synthesis of well-stabilized Au NPs using potato extract. The association of the SPR peak at 520 nm, together with the uniform nanoscale morphology, indicated effective bio-reduction and capping by phytochemical constituents. These characteristics make the biosynthesized nanoparticles promising for further applications in catalysis, sensing, and biomedical fields, where particle size and colloidal stability are critical factors [2].

Pregnancy is a complicated biological process, including many developmental stages of the fetus. Body weight loss is a signal of systemic toxicity [39]. Interestingly, treated mice exhibited a slight increase in body weight, potentially attributable to the low toxicity of the biosynthesized Au NPs formulation used [40]. However, more sensitive endpoints revealed significant developmental toxicities. A marked, dose-dependent increase in fetal resorption was observed in treated groups compared to controls. This suggests that Au NPs exposure induces complications in pregnancy, likely through mechanisms that impair the maternal-fetal interface [12]. To understand this fetotoxicity, the unique placental anatomy of the murine model must be taken into account. Unlike humans, the mouse placenta incorporates a significant yolk sac component, which is critical for early nutrient transport before the chorioallantois placenta is fully established [41].

Furthermore, the definitive mouse placental labyrinth consists of three trophoblast layers, forming a densely branched structure that differs from the human villous tree. These anatomical distinctions are crucial for interpreting nanoparticle transfer. The potential accumulation of Au NPs in both the yolk sac and the placental labyrinth likely governs their distribution to the fetus, and structural differences may directly influence the efficiency and pathway of NP uptake [42].

In the present study, the increase in fetal weight in the groups treated with Au NPs was accompanied by a parallel increase in the ossified long bones. This is consistent with **Sucheston** et al. [43], who found a positive correlation between ossified long-bone measurements and fetal weight after treating mice maternally with anticonvulsant drugs. However, this result contrasts with **Sun** et al. [44], who observed that mice exposed to nanoparticles via the maternal route showed decreased fetal growth and increased fetal mortality.

The present results show that Au NPs help in the development of the skull bones according to an increase in red coloration in the process of osteogenesis. The skull bones that revealed advances in ossification were parietal, interparietal, tympanic bulla, squamosal, periotic, supraoccipital, palatine, pterygoid, and ethmoid bones. These results are in contrast with **Ungewitter** et al. [45], who observed skeletal delayed ossification in the components of the skull of mouse fetuses maternally treated with di-(2-ethylhexyl) phthalate (DEHP). It has been shown that the skull shapes of the three treated groups were approximately the same as those of the control. In the components of the second and third groups, the skull is stained red without blue, indicating that the ossification was dilated due to treatment with Au NPs. This result is considered a positive result.

The present investigations indicate that abnormal ossification of the vertebrae and curvature are associated with a higher Au NPs dose in the third group. These observations are consistent with **Shim** et al. [46], who found severe vertebral column malformations in COX-2 transgenic fetuses. Similarly, **Fadel** et al. [47] reported abnormal rib ossification in mouse fetuses maternally treated with therapeutic doses of topiramate. These defects appeared in the elongation of the sternebra, dislocation of the cartilaginous ribs, and cleavage of the last sternebra. Comparable abnormalities in the sternum were also reported by **Ungewitter** et al. [45] in mouse fetuses maternally treated with DEHP.

Genetic and environmental factors cause skeletal variations. Chondrogenesis is the initial step in embryonic skeletal development. This process is associated with signaling events that regulate gene transcription and function. Chondrogenesis is a regulated, multistep process in which chondrocytes proliferate and differentiate during formation. The expression of specific genes characterizes this. Type II collagen is found in early proliferative chondrocytes and is essential for cartilage extracellular matrix formation [46, 48, 49]. The type X collagen gene is expressed in hypertrophic chondrocytes and regulated by RUNX2. RUNX2 is a known master transcription factor of maturing chondrocytes and developing osteoblasts [50]. Cell proliferation plays a significant role in normal limb development. Cell proliferation is responsible for bone elongation and limb outgrowth. Our observation indicated that Au NPs enhance cell proliferation. In addition, these observations suggest that exposure to Au NPs increased the rate of matrix mineralization and ossification in the limbs. This result is in contrast with **Bigaeva** et al. [48], who suggested that a high concentration of quantum dots carrying mercaptopropionic acid (QD-MPA) showed an inhibitory effect on type II and type X collagen expression, as well as caused defects or delays in the processes of matrix mineralization and ossification in limbs.

In general, bone development depends on many factors, including hormones, nutrition, genes, environment, and physical activity [51, 52]. The mechanism involved in bone development was based on Frost’s mechanistic theory [53]. The mechanical requirements of the bone coordinate the bone cell activity. Moreover, when the mechanical challenge exceeds a suitable threshold, bone tissue is added at the location. Fetal physical activity occurs through regular fetal kicks against the uterine wall. Since Au NPs cross the placenta and accumulate in fetal tissue, increased fetal physical activity may lead to dilated ossification and increased bone formation.

Some studies have demonstrated the importance of a class of conserved genes expressed during early morphogenesis. This class of genes, known as homeobox (Hox) genes, is expressed in the segmented mesoderm that gives rise to the vertebral column. The expression of homeobox genes appears to be correlated with the differentiation of developing skeletal systems, including the limbs, head, and vertebral axis. Treatment with Au NPs may increase the *Hox* gene expression and result in the elongation of vertebral identities. *Hox* gene expression may regulate local growth timing and rates in developing vertebrae, thereby producing any of the morphological features that characterize individual vertebrae [53, 54].

The multimodal interpretation of bone ossification presented in this study integrates results obtained from both LIBS and laser speckle imaging. LIBS measurements provide elemental information related to bone ossification and mineral density. The results demonstrate that increasing sample ossification increases the shock-wave propagation velocity within the plasma. The accelerated shock wave enhances plasma compression, thereby elevating the excitation temperature. This increase in temperature promotes greater ionization efficiency, producing stronger ionic emission lines and a higher ionic-to-atomic spectral line intensity ratio. Consistent with this behavior, **Abdel-Salam** et al. [55] and **Galmed** et al. [22] demonstrated a direct correlation between surface hardness and shock wave velocity, confirming that ossified materials generate hotter plasmas with enhanced ionic-to-atomic ratios.

Regarding laser speckle imaging, it offers a sensitive, non-destructive tool for probing microstructural changes associated with bone ossification through speckle-derived parameters and SMI. Strong negative correlations between global contrast and entropy with the level of ossification suggest that as mineralization proceeds, heterogeneity in scattering complexity decreases. On the other hand, the moderate positive correlation between the BSD Slope and ossification indicates that the spatial frequency content of the speaker pattern shifts toward lower frequencies as the tissue matrix becomes optically smoother. These findings, together, support the idea that speckle-based metrics capture meaningful changes in optical scattering linked to underlying microstructural evolution during bone development. Therefore, the SMI, combining these complementary parameters, provides a robust optical measure for quantifying ossification and mineral content. In summary, the combined analysis enables a comprehensive assessment of Au NPs-induced skeletal changes at the compositional and microstructural levels.

## Conclusion

This study demonstrates that intravenously administered biosynthesized Au NPs cross the placental barrier in a BALB/c mouse model and induce apparent, dose-dependent fetotoxic effects. These effects were characterized by using a complementary dual-analytic approach, including LIBS for high-sensitivity elemental analysis and laser speckle imaging for noninvasive microstructural assessment. Disrupted ossification patterns altered skeletal microarchitecture, and significant imbalances in key bone elements (notably calcium and magnesium) have been detected. These combined photonic and spectroscopic approaches revealed subtle elemental and morphological defects that escaped conventional histology, underscoring their superior sensitivity for early detection of developmental toxicity. Beyond confirming that transplacental accumulation of Au NPs can induce skeletal anomalies, our results validate an integrated, multimodal workflow as a powerful tool for nanotoxicological evaluation. It is recommended that advanced spectroscopic and imaging methods be incorporated into preclinical safety pipelines to capture subvisual and elemental-level impacts during vulnerable developmental windows. Further mechanistic studies, extended dose-response and longitudinal assessments, and standardized protocols are needed to inform safe clinical translation and regulatory guidance for nanomaterials intended for biomedical use.

## Data Availability

Data will be made available on request.

## Abbreviations

Au NPs: Gold Nanoparticles
LIBS: Laser-Induced Breakdown Spectroscopy
SMI: Speckle Microstructure Index
KDE: Kernel Density Estimation
Na: Nasal
Fro: Frontal
P: Parietal
IP: Interparietal
Sq: Squamosal
B.oc: Basioccipital
T.V.: Thoracic Vertebrae
L.V: Lumbar Vertebrae
S.V.: Sacral Vertebrae
C.V: Cervical Vertebrae
PMX: Premaxilla
Zy. P. J.: Zygomatic Process of Jugal
BO.: Basioccipital
EO: Exoccipital
T. B: Tympanic Bulla
PS: Presphenoid
BS.: Basisphenoid
